# Respiratory Oscillometry in Newborn Infants: Conventional and Intra-Breath Approaches

**DOI:** 10.3389/fped.2022.867883

**Published:** 2022-04-04

**Authors:** Bence L. Radics, Zita Gyurkovits, Gergely Makan, Zoltán Gingl, Dorottya Czövek, Zoltán Hantos

**Affiliations:** ^1^Department of Pathology, University of Szeged, Szeged, Hungary; ^2^Department of Obstetrics and Gynecology, University of Szeged, Szeged, Hungary; ^3^Department of Technical Informatics, University of Szeged, Szeged, Hungary; ^4^1st Department of Pediatrics, Semmelweis University, Budapest, Hungary; ^5^Department of Anesthesiology and Intensive Therapy, Semmelweis University, Budapest, Hungary

**Keywords:** infant oscillometry, respiratory resistance, respiratory reactance, respiratory compliance, nasal resistance, intra-breath method

## Abstract

**Background:**

Oscillometry has been employed widely as a non-invasive and standardized measurement of respiratory function in children and adults; however, limited information is available on infants.

**Aims:**

To establish the within-session variability of respiratory impedance (Zrs), to characterize the degree and profile of intra-breath changes in Zrs and to assess their impact on conventional oscillometry in newborns.

**Methods:**

109 healthy newborns were enrolled in the study conducted in the first 5 postpartum days during natural sleep. A custom-made wave-tube oscillometry setup was used, with an 8–48 Hz pseudorandom and a 16 Hz sinusoidal signal used for spectral and intra-breath oscillometry, respectively. A resistance-compliance-inertance (R-C-L) model was fitted to average Zrs spectra obtained from successive 30-s recordings. Intra-breath measures, such as resistance (Rrs) and reactance (Xrs) at the end-expiratory, end-inspiratory and maximum-flow points were estimated from three 90-s recordings. All natural and artifact-free breaths were included in the analysis.

**Results:**

Within-session changes in the mean R, C and L values, respectively, were large (mean coefficients of variation: 10.3, 20.3, and 26.6%); the fluctuations of the intra-breath measures were of similar degree (20–24%). Intra-breath analysis also revealed large swings in Rrs and Xrs within the breathing cycle: the peak-to-peak changes amounted to 93% (range: 32–218%) and 41% (9–212%), respectively, of the zero-flow Zrs magnitude.

**Discussion:**

Intra-breath tracking of Zrs provides new insight into the determinants of the dynamics of respiratory system, and highlights the biasing effects of mechanical non-linearities on the average Zrs data obtained from the conventional spectral oscillometry.

*Motto:*

*“Accurate assessment of lung function in infants is no mean undertaking – requiring not only the highest specifications from equipment …but infinite patience and meticulous attention to detail from the operators.”*

*Janet Stocks, 2004*

*Pediatric Anesthesia 14: 537–540*

## Introduction

The burdens of infant pulmonary function testing (PFT) imposed by the lack of active cooperation, the requirement for sleep, the obligatory nasal breathing, the high impedance of the respiratory system and several other factors have prevented the establishment of a gold standard in infant PFT. Respiratory oscillometry measures the mechanical impedance of the respiratory system (Zrs), and it has been shown as a promising method in different measurement settings (1). Recent work has demonstrated its feasibility in normally breathing unsedated infants with a high success rate (2–5). Additionally, a new tracking modality of oscillometry (6) has revealed disease-specific patterns of intra-breath changes in Zrs and has proven unique in predicting lower respiratory tract illness during infancy (7). However, a comprehensive analysis is still needed to fully characterize the intra-breath dynamics of Zrs in infants, with special regard to the substantial contribution of the upper airways (8–12). Confrontation of the novel intra-breath oscillometry with conventional spectral oscillometry is also lacking. While some data on the day-to-day Zrs changes in newborn infants are available (5, 13), the within-session reproducibility of oscillometry measures has not been studied.

The aims of the present study were (a) to measure Zrs in healthy term newborns to characterize the physiological flow (V’)- and volume (V)-dependent changes *via* intra-breath oscillometry, (b) to examine the potentially confounding effects of intra-breath changes on average Zrs spectra obtained from conventional multi-frequency measurements and (c) to determine the within-session variability of conventional and intra-breath oscillometry variables.

## Materials and Methods

The study protocol was approved by the Institutional Clinical Ethics Committee of the University of Szeged (91/2011, renewed in 2017). A written informed consent and assent was obtained from all mothers prior to the subject recruitment. The data collection period started in January 2017 and ended in May 2017. All measurements were performed in the Neonatal Unit, Department of Obstetrics and Gynecology, University of Szeged.

Healthy term infants (> 37th week of gestation, birthweight > 2,500 g, APGAR score at 5 min ≥ 8, uninterrupted early adaptation) were included in the study. Lung function was measured between the 2nd–5th postpartum day on a single occasion, during natural sleep. Newborns were excluded from the study if steady-state breathing was not reached or leakage persisted around the face mask despite multiple trials.

### Measurement Setup

Oscillometric measurement of input Zrs was made with a custom-made wave-tube setup (length: 20 cm, internal diameter: 8 mm), in a setting similar to that described previously (5, 7). Small-amplitude (0.5 hPa) oscillations were generated by the loudspeaker and superimposed on the breathing. Spectral oscillometric recordings were 30 s long, and five different pseudorandom signal specimens containing components at every 4 Hz between 8 and 48 Hz were applied. Intra-breath oscillometric recordings lasted for 90 s, and a single 16 Hz sinusoid was used. Multiple measurements were performed with both modalities in random order, without removing the face mask between recordings if the sleep stage was uninterrupted.

Airflow (V’) was measured with a custom-made pneumotachograph. The wave-tube and the pneumotachograph were equipped with identical pressure sensors (Honeywell model 26PCAFA6D, Golden Valley, MN, United States). Single-use bacterial filter (Gibeck, Humid-Vent filter, small straight type, No. 19502 Teleflex Medical, Athlone, Ireland) and face mask (Hudson RCI, air-cushion mask with inflation valve, neonate size, No. 41277, Teleflex Medical) were attached to the setup. The equipment’s dead space was flushed by medical air at a rate of 2 L.min^–1^ to avoid hypercapnia.

Transcutaneous monitoring of peripheral hemoglobin oxygen-saturation was done (Edan M50, Bell Medical, Inc., St. Louis, MO, United States) during the recordings for safety reasons. No desaturation episode was detected during data collection. Oxygen saturation data were not stored for further analysis.

### Signal Processing

Pressure and V’ signals were sampled at a rate of 512 s^–1^, bandpass filtered in the 4–50 Hz range for spectral oscillometry and the 14–18 Hz range for the intra-breath measurements. Zrs was calculated based on the auto- and cross-correlation spectra of the wave-tube’s lateral pressures using the fast Fourier transform, and expressed as resistance (Rrs) and reactance (Xrs). The intra-breath Zrs values were computed for each oscillation cycle (0.0625 s) and a moving average was calculated over a time window of 0.25 s. The signals of volume (V) and volume acceleration (V”), respectively, were obtained by numerical integration and differentiation of V’.

### Analysis of Zrs Spectra

An average spectrum was calculated from a minimum of 3 recordings of lowest Rrs. Recordings or segments thereof containing artifacts, such as glottis closure, vocalization, body movements and leaks around the mask were discarded. No criteria relating to tidal volume (V_T_) were set and sighs *per se* were not considered as artifacts. A simple resistance (R)—compliance (C)—inertance (L) model (14) was fit to the average Zrs data, as described in detail previously (5). Conventional spectral oscillometry measures, such as the lowest-frequency (8-Hz) values of Zrs magnitude (|Z_8_|), resonance frequency (f_res_) and reactance area below f_res_ (Ax) were also calculated; the frequency dependence of Rrs was characterized by the difference in Rrs between 8 Hz and 32 Hz (R_8–32_).

### Intra-Breath Measures

**FIGURE 1:**
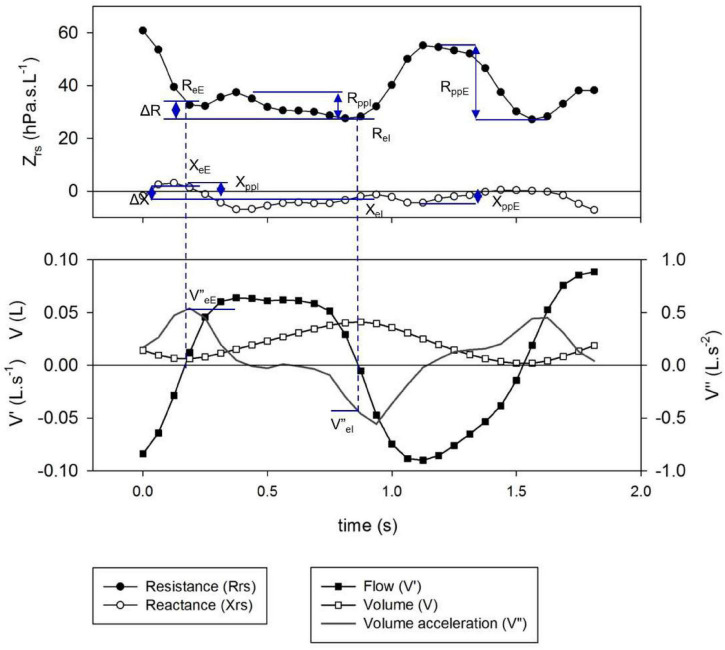
Definition of specific intra-breath measures of resistance (Rrs) and reactance (Xrs). Shown are the time points of end inspiration (eI)- and end expiration (eE) (indicated by dotted lines), their differences (Δ) and peak-to-peak changes in inspiration (ppI) and expiration (ppE).

All regular artifact-free breaths (see previous section) except sighs were included in the analysis. Specific points of the respiratory cycle were selected to characterize the intra-breath dynamics of Zrs (Figure 1). Values of Rrs at end-expiration and end-inspiration (R_eE_ and R_eI_, respectively) were calculated from the closest data points to zero V’ obtained with linear interpolation. Tidal change in Rrs (ΔR) was determined as R_eE_-R_eI_. Peak-to-peak changes in Rrs during inspiration (R_ppI_) and expiration (R_ppE_) were determined. The corresponding parameters of Xrs (X_eE_, X_eI_, ΔX, X_ppE_ and X_ppI_) and the average zero-flow impedance magnitude, |Z_0_| = |1/2(Z_eE_ + Z_eI_)| were also calculated.

### Tidal Breathing Parameters

Simple tidal breath descriptors, such as V_T_, respiratory rate (f_br_), ratio of expiratory time over cycle time (T_E_/T_tot_), and the ratio of time to peak expiratory flow (V’_maxE_) and T_E_ (T_PTEF_/T_E_) were obtained from the spirogram. Volume acceleration at end-expiration and end-inspiration (V”_eE_ and V”_eI_, respectively) were determined from pairs of V” data adjacent to the zero crossing.

### Statistical Analysis and Graphics

Data are presented as mean ± standard deviation (SD). Two sample *t*-test, correlation analysis with Pearson’s correlation coefficients were performed with the open-source RStudio software^1^ based on R language (R.4.1). Cluster analysis was also performed in R using Euclidean distances and Ward’s hierarchical method. Graphs were prepared with SigmaPlot 13.5 (Systat Software Inc., San José, CA, United States).

## Results

A total of 109 newborns were enrolled in the study. Six subjects were excluded due to technical reasons (see pre-defined exclusion criteria in the “Materials and Methods” section). Although the measurements were technically acceptable, 17 of the remaining 103 subjects were excluded on the basis of physiologically unrealistic values of Zrs parameters, such as negative L (*n* = 4), low C (< 0.5 mL.hPa^–1^) (*n* = 6) or high RL product (>10 hPa^2^.s^3^.L^–2^) suggestive for nasal obstruction (*n* = 7); in 4 of these 17 subjects, two exclusion criteria applied. Most of these subjects were also identified as outliers during regression diagnostics, and therefore they were omitted from further analysis. Statistical analysis was performed on the data of the remaining 86 newborns (41 females, 45 males; spontaneous delivery: 41, caesarean section: 45) whose anthropometric characteristics are summarized in Table 1.

**TABLE 1:** Comparison of anthropometry and spirogram data between subject groups of different patterns of flow dependence of reactance.

	All (*n* = 86)	Pattern A (*n* = 47)	Pattern B (*n* = 27)	Pattern C (*n* = 5)	Pattern D (*n* = 7)
GA (weeks)	38.7 ± 1.3	38.9 ± 1.2	38.6 ± 1.2	38.0 ± 2.1	38.3 ± 1.4
BL (cm)	49.5 ± 2.4	50.0 ± 2.5	49.3 ± 2.3	48.0 ± 2.7	47.7 ± 1.5**
BW (g)	3,269 ± 546	3,365 ± 569	3,293 ± 490	2,694 ± 491*	2,931 ± 210**
f_br_ (min^–1^)	62.0 ± 11.4	65.3 ± 12.2	58.2 ± 9.5**	57.9 ± 5.0*	57.5 ± 10.1
V_T_ (mL)	29.3 ± 5.5	29.3 ± 5.9	29.7 ± 4.6	26.2 ± 3.6	30.6 ± 6.5
V’_maxE_ (mL.s^–1^)	90 ± 17	93 ± 17	81 ± 15**	77 ± 15	81 ± 16
T_E_/T_tot_	0.50 ± 0.03	0.50 ± 0.03	0.52 ± 0.03**	0.47 ± 0.03	0.51 ± 0.01
T_PTEF_/T_E_	0.47 ± 0.07	0.47 ± 0.07	0.45 ± 0.09	0.49 ± 0.07	0.49 ± 0.07
CoV[T_tot_]	0.155 ± 0.058	0.155 ± 0.059	0.168 ± 0.055	0.132 ± 0.032	0.118 ± 0.058
CoV[V_T_]	0.219 ± 0.092	0.231 ± 0.07	0.212 ± 0.069	0.162 ± 0.072	0.202 ± 0.097

*Mean ± SD values.*

*Pattern A: minimal dependence of reactance (Xrs) on flow (V’).*

*Pattern B: marked V’-dependent decrease in Xrs during expiration.*

*Pattern C: marked V’-dependent decrease during inspiration.*

*Pattern D: marked V’-dependent increases in Xrs.*

*GA, gestational age; BL, birth length; BW, birth weight; f_br_, respiratory rate; V_T_, tidal volume; V’_maxE_, peak expiratory flow; T_E_, expiratory time; T_tot_, total respiratory cycle time; T_PTEF_, time to peak tidal expiratory flow; CoV, coefficient of variation.*

*p < 0.05 vs. Pattern A.

**p < 0.01 vs. Pattern A.

The mean total recording time in the 103 subjects was 14 min (range: 8–21 min); the recordings were suspended for 3–10 min in 13 subjects, and the measurements were successful only on the following day in 3 neonates. On the average, 48 (range: 15–105) respiratory cycles were analyzed from the intra-breath oscillometry in each newborn; these were collected as segments of steady-state breathing from a minimum of three 90-s recordings. The average values of spectral outcomes were calculated from 6 (3–11) recordings of a mean length of 26 s (12–30 s).

Overall, the intra-breath changes in Zrs, dominated by the V’ dependence, were remarkably large. R_ppE_ and R_ppI_ amounted to 91.4 ± 33.3% and 55.9 ± 27.6%, respectively, of the average zero-flow impedance magnitude, i.e., Z_0_ = 1/2(Z_eE_ + Z_eI_). The maximum Rrs was usually located near the peak V’, while the minimum was found around V’ = 0. The corresponding changes in Xrs (X_ppE_ and X_ppI_) were roughly half as large (44.9 ± 26.8% and 32.7 ± 19.3%, respectively). Tidal change in Rrs was on the average close to zero (ΔR = −0.4 ± 6.5 hPa.s.L^–1^), with negative values of ΔR were observed in 51% of the subjects, whereas the decreases in Xrs between end expiration and end inspiration were more uniform (ΔX = 2.39 ± 3.44 hPa.s.L^–1^).

**FIGURE 2:**
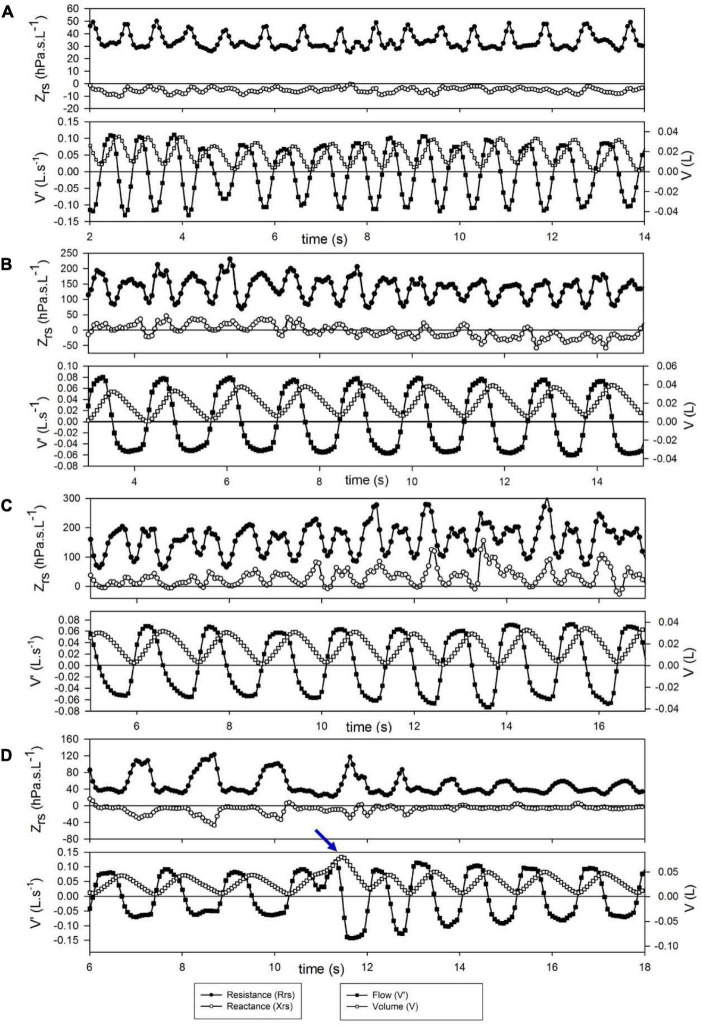
Examples of short term changes in impedance (Zrs) and breathing pattern. Each graph represents a 12-s period. **(A)** Slightly irregular tidal flow but stable and low Zrs. **(B)** Slow downward drift in reactance (Xrs) during regular breathing. **(C)** Increasing flow dependence of Zrs during steady-state breathing; this probably reflects spontaneous development of nasal obstruction. **(D)** Transient decrease of expiratory flow limitation after a spontaneous sigh (arrow).

Short-term changes in Zrs are illustrated with a few selected segments of intra-breath recordings (Figure 2). These examples are not intended to be exhaustive; they only highlight epochs where (i) regular intra-breath fluctuations in Rrs and Xrs are observed despite a slightly irregular spirogram (Figure 2A), (ii) a slow negative drift in Xrs occurs (Figure 2B) or (iii) increasing fluctuations in both Rrs and Xrs take place (Figure 2C) at virtually even tidal volumes, and (iv) the large expiratory increases in Rrs and decreases in Xrs are reduced following a sigh (Figure 2D).

**FIGURE 3:**
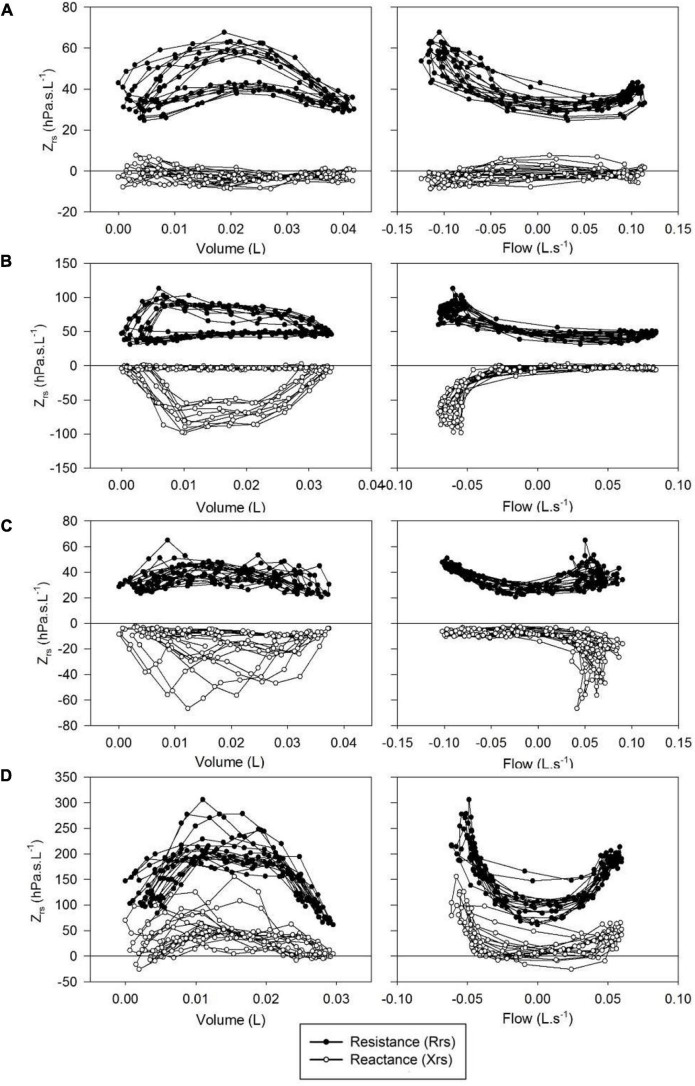
Typical patterns of intrabreath changes in respiratory impedance (Zrs). **(A)** Insignificant changes in reactance (Xrs) and mild flow non-linearity in resistance (Rrs); **(B)** marked fall in Xrs and increase in Rrs during expiration; **(C)** marked fall in Xrs in inspiration; **(D)** increases in Xrs and Rrs with both inspiratory and expiratory flow.

**FIGURE 4:**
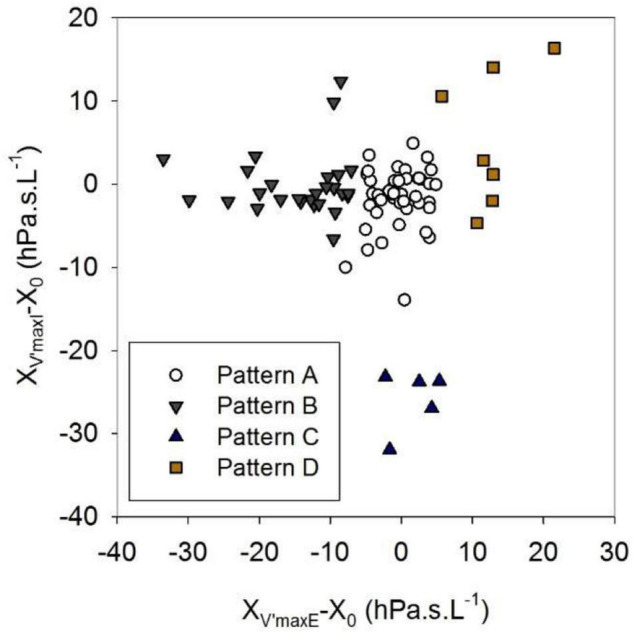
Clusters established in the relationships between inspiratory and expiratory flow dependences of reactance. X_V’maxI_ and X_V’maxE_ are reactance values at peak inspiratory and expiratory flows, X_0_ is the average zero-flow value of reactance. For definitions of Patterns A-D, see text or the legend to Table 1.

Whereas Rrs exhibited positive V’ dependences during inspiration and expiration, the intra-breath changes in Xrs were more diverse. Four typical patterns were determined qualitatively and are exemplified in Figure 3 where Rrs and Xrs are plotted against V and V’. These patterns are characterized as minimal dependence of Xrs on V’ (Pattern A), marked V’-dependent decrease in Xrs during expiration (Pattern B), marked V’-dependent decrease during inspiration (Pattern C) and marked V’-dependent increases in Xrs (Pattern D). Each newborn was classified into one group according to the V’-dependence of Xrs by cluster analysis, presuming that four different patterns exist (Figure 4). Subjects with the lowest V’-dependence in Xrs (Pattern A) were considered the control group. Tables 1–3, respectively, contain the anthropometrical and tidal breathing data, the spectral oscillometry measures and the intra-breath variables in the 4 clusters. Slightly lower body measures were found in the Pattern C and D groups and lower f_br_ values in Pattern B-D groups compared to the Pattern A data (Table 1). L was the highest while f_res_ and Ax were the lowest in the positive V’ dependence (Pattern D) group (Table 2). L was significantly (*p* < 0.05) lower and Ax was higher in subjects with negative expiratory swings in Xrs (Pattern B) compared to Pattern A. Unlike the values of X_8_, parameter C was found to be not different between groups. The overall fitting error of the R -C-L model to the Zrs data was 7.4 ± 2.8%; its components broken down to Rrs and Xrs were 6.3 ± 2.7% and 3.7 ± 1.6%, respectively.

**TABLE 2:** Comparison of spectral oscillometry data between subject groups of different patterns of flow dependence of respiratory reactance.

	All (*n* = 86)	Pattern A (*n* = 47)	Pattern B (*n* = 27)	Pattern C (*n* = 5)	Pattern D (*n* = 7)
R (hPa.s.L^–1^)	48.7 ± 12.9	46.0 ± 12.6	48.7 ± 11.5	59.9 ± 17.4	58.3 ± 9.4*
C (mL.hPa^–1^)	1.08 ± 0.30	1.13 ± 0. 32	1.01 ± 0. 29	1.05 ± 0.28	1.06 ± 0. 15
L (hPa.s^2^.L^–1^)	0.068 ± 0.028	0.071 ± 0.027	0.057 ± 0.023*	0.047 ± 0.031	0.102 ± 0.020**
R_8_ (hPa.s.L^–1^)	63.2 ± 16.8	59.6 ± 16.3	64.6 ± 16.4	77.3 ± 22.1	72.0 ± 11.2*
X_8_ (hPa.s.L^–1^)	−14.9 ± 5.4	−13.9 ± 5.1	−16.7 ± 5.8*	−17.1 ± 5.8	−13.3 ± 4.2
R_8–32_ (hPa.s.L^–1^)	18.6 ± 7.3	16.9 ± 6.4	20.6 ± 8.5	24.7 ± 5.9*	17.6 ± 5.0
f_res_ (Hz)	21.4 ± 5.9	20.1 ± 5.2	23.7 ± 4.7**	29.2 ± 11.9	16.3 ± 1.9**
Ax (hPa.L^–1^)	103.1 ± 59.6	90.7 ± 55.1	124.8 ± 58.5*	157.0 ± 80.3	64.0 ± 20.8*

*Mean ± SD values. For definitions of Patterns A-D, see text or the legend to Table 1. R, resistance (model fitting); C, compliance (model fitting); L, inertance (model fitting); R_8_, resistance at 8 Hz; X_8_, reactance at 8 Hz; R_8–32_, resistance difference between 8 and 32 Hz; f_res_, resonance frequency; Ax, reactance area below f_res_.*

*p < 0.05 vs. Pattern A; **p < 0.01 vs. Pattern A.

**TABLE 3:** Comparison of intra-breath oscillometry data between subject groups of different patterns of flow dependence of respiratory reactance.

	All (*n* = 86)	Pattern A (*n* = 47)	Pattern B (*n* = 27)	Pattern C (*n* = 5)	Pattern D (*n* = 7)
R_eE_ (hPa.s.L^–1^)	41.7 ± 11.3	38.7 ± 10.1	41.9 ± 9.0	53.3 ± 16.0	52.7 ± 13.2*
R_eI_ (hPa.s.L^–1^)	42.1 ± 13.5	38.5 ± 13.4	43.7 ± 11.7	51.3 ± 15.8	53.5 ± 11.2*
X_eE_ (hPa.s.L^–1^)	−1.35 ± 3.98	−1.55 ± 3.55	−1.516 ± 3.14	−6.74 ± 2.84*	4.53 ± 3.86**
X_eI_ (hPa.s.L^–1^)	−3.73 ± 4.21	−3.14 ± 3.54	−5.45 ± 3.69*	−8.05 ± 4.23	1.92 ± 3.92*
ΔR (hPa.s.L^–1^)	−0.40 ± 6.48	0.23 ± 6.30	−1.83 ± 7.48	2.03 ± 2.43	−0.81 ± 5.16
ΔX (hPa.s.L^–1^)	2.39 ± 3.44	1.58 ± 3.30	3.93 ± 3.72**	1.30 ± 1.74	2.62 ± 2.36
R_ppE_/|Z_0_|	0.91 ± 0.33	0.80 ± 0.26	1.08 ± 0.32**	0.67 ± 0.20	1.25 ± 0.43*
R_ppI_/|Z_0_|	0.56 ± 0.28	0.52 ± 0.24	0.50 ± 0.22	0.87 ± 0.21*	0.85 ± 0.46
X_ppE_/|Z_0_|	0.45 ± 0.27	0.32 ± 0.13	0.70 ± 0.31**	0.28 ± 0.08	0.50 ± 0.14**
X_ppI_/|Z_0_|	0.32 ± 0.19	0.30 ± 0.13	0.28 ± 0.09	0.90 ± 0.28**	0.33 ± 0.14

*Mean ± SD values. For definitions of Patterns A–D, see text or the legend to Table 1. R_eE_, resistance at end expiration; R_eI_, resistance at end inspiration; X_eE_, reactance at end expiration; X_eI_, reactance at end inspiration; ΔR, tidal change in resistance (R_eE_-R_eI_); ΔX, tidal change in reactance (X_eE_-X_eI_); R_ppE_, peak-to-peak resistance difference in expiration; R_ppI_, peak-to-peak resistance difference in inspiration; X_ppE_, peak-to-peak resistance difference in expiration; X_ppI_, peak-to-peak resistance difference in inspiration; |Z_0_|, impedance magnitude at zero flow [1/2(|Z_eE_| + |Z_eI_|)].*

*p < 0.05 vs. Pattern A; **p < 0.01 vs. Pattern A.

Comparison of intra-breath measures (Table 3) revealed mild elevations in R_eE_ and R_eI_ in the C and D groups but no differences in ΔR between the different patterns. ΔX reached significantly higher values in the Pattern B group than in the rest of groups. Differences in the V’ dependence of Rrs measures (R_ppE_ and R_ppI_) were milder between groups than that of Xrs measures (X_ppE_ and X_ppI_) as the latter are related to the clustering variables (Figure 4).

**FIGURE 5:**
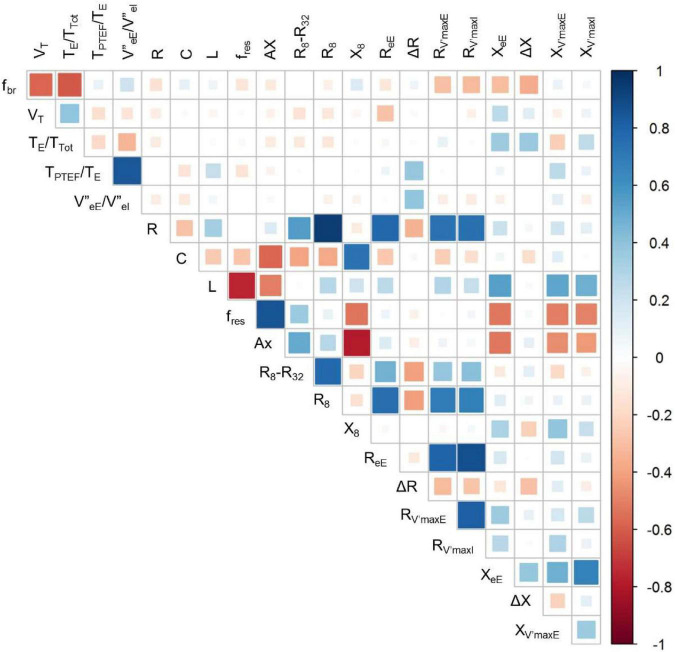
Correlogram for selected measures of tidal breathing, spectral oscillometry and intra-breath oscillometry. For definition of variables, see legend to Table 4.

**FIGURE 6:**
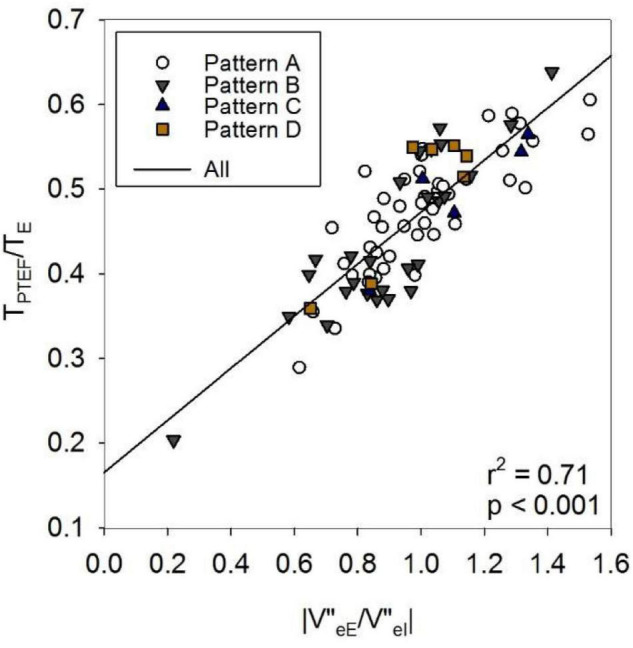
Effect of asymmetry in volume acceleration (V”) ratio on T_PTEF_/T_E_. eE: end expiration; eI: end inspiration; T_PTEF_: time to peak tidal expiratory flow; T_E_: expiratory time. For definitions of Patterns A–D, see text or the legend to Table 1.

Figure 5 gives an overview on the correlations between selected indices of the spirogram, spectral oscillometry and intrabreath analysis. Among the between-category comparisons, high correlation coefficients were found between R and the intra-breath Rrs measures and between the spectral (L, f_res_ and Ax) and intra-breath Xrs measures, except C which was most correlated with Ax and X_8_ but not with intra-breath Xrs data. A weak although statistically significant (*r* = 0.39, *p* < 0.001) linear correlation was found between ΔR and |V”_eE_/V”_eI_| . T_PTEF_/T_E_ was not correlated with any of the spectral and intra-breath Rrs or Xrs outcomes, but exhibited a very strong relationship (*r* = 0.84, *p* < 0.001) with |V”_eE_/V”_eI_|, apparently unrelated to the pattern of V’ dependence of Xrs (Figure 6).

**TABLE 4:** Within-session variability presented as coefficient of variation (in%) of tidal breathing, spectral oscillometry and intra-breath oscillometry indices.

f_br_	14.0 (3.9–38.2)
V_T_	21.9 (7.8–42.3)
T_E_/T_tot_	7.9 (3.3–13.8)
T_PTEF_/T_E_	19.6 (11.1–44.4)
R	10.3 (1.9–29.7)
C	20.3 (3.3–88.4)
L	26.6 (3.6–154.0)
|Z_8_|	13.6 (5.3–30.7)
R_8–32_	26.5 (5.2–64.6)
f_res_	15.7 (1.9–67.6)
Ax	37.9 (7.5–186.1)
|Z_eE_|	19.6 (5.3–117.7)
|Z_eI_|	23.1 (4.9–100.6)
|Z_V’maxE_|	23.6 (4.3–71.0)
|Z_V’maxI_|	22.2 (5.5–58.0)

*Mean (range) values. f_br_, respiratory rate; V_T_, tidal volume; V’_maxE_, peak expiratory flow; T_E_, expiratory time; T_tot_, total respiratory cycle time; T_PTEF_, time to peak tidal expiratory flow; R, resistance (model fitting); C, compliance (model fitting); L, inertance (model fitting); |Z_8_|, impedance magnitude at 8 Hz; R_8–32_, resistance difference between 8 and 32 Hz; f_res_, resonance frequency; Ax, reactance area below f_res_.; |Z_eE_|, impedance magnitude at end expiration; |Z_eI_|, impedance magnitude at end inspiration; |Z_V’maxE_|, impedance magnitude at maximum expiratory flow; |Z_V’maxI_|, impedance magnitude at maximum inspiratory flow.*

## Discussion

The 94% success rate in the present study confirms earlier observations on the feasibility of oscillometry in unsedated newborns (5) and infants (2, 4, 7) although its outstanding value can largely be attributed to the favorable environmental and time allocation circumstances in the neonatal ward. These factors enabled a more detailed assessment of short-term variability of Zrs in healthy term neonates.

### Intra-Breath Changes in Rrs

The characteristic effect of V’ on Rrs was documented in early studies using single-frequency oscillations in orally breathing adult subjects (15, 16). These biphasic changes in Rrs, characterized by minimum values at zero V’ and local maxima at peak inspiratory and expiratory V’ (V’_maxI_ and V’_maxE_, respectively) were a marked feature in the neonates of this study, with the non-linearity in expiration usually exceeding that of inspiration. Previous observations suggest that the non-linear, V’-dependent increase in Rrs originates from the upper airways (12, 17, 18), obeying the classical empirical description by Rohrer (19).

An unexpected finding in the present investigation was the fact that R_eI_ was higher than R_eE_ (i.e., ΔR was negative) in almost half of the subjects. This is in contrast to previous intra-breath studies where the typically positive ΔR values were attributed to the tidal dilatation of the pulmonary airways (7, 20, 21). One important specific factor in infancy is the large contribution of the extrathoracic pathways to Rrs, whose transmural pressures are dependent on V’ rather than V and are opposite to that of the pulmonary airways; this may lead to narrowing of the upper airways during inspiration and possibly some residual constriction at end inspiration. Another factor, also augmented in nasal breathing is the non-steady flow patterns that develop at fast transitions of V’ in the upper airways of irregular geometry. This leads to extra dissipation, which has been shown to depend on the rate of change in V’ (i.e., on V”) (12, 17, 18), and would add to the true “zero-flow” values of R_eE_ and R_eI_. In the present study, the asymmetry of respiratory phase change (as characterized by the ratio V”_eE_/V”_eI_) was shown to correlate with ΔR. Since the transition from inspiration to expiration is usually faster than *vice versa*, it can lead to low, or even negative values in ΔR. These factors discussed above suggests that the contributions of the upper airway to ΔR may mask the change in pulmonary airway caliber. Nevertheless, the near-zero mean value of ΔR is at variance with the results of the intra-breath measurements in infants (7) where an average of 4.43 hPa.s.L^–1^ (IQR: 0.65–8.13 hPa.s.L^–1^) was observed. Since the same custom-made wave-tube device was employed in both studies and the spectral Zrs measures are similar, differences between the 2 populations, such as ethnic (Caucasians vs. Black Africans), age (newborns vs. 6 week old infants), gestational age (term vs. term + late preterm) and other characteristics may explain the different ΔR values.

### Intra-Breath Changes in Xrs

While the changes in Rrs within the respiratory cycle are dominated by the “U” shape in V’ dependence of different degrees and asymmetry, Xrs exhibited qualitatively more distinct intra-breath patterns. We defined a group with the lowest V’-dependent changes in Xrs (pattern A) and considered it the control group. The rest (45%) of the examined neonates exhibited diverse and strong V’ dependences of Xrs. Three additional typical V’-dependent patterns were identified qualitatively and verified by cluster analysis. Inference to the underlying mechanisms of each pattern is burdened by the lack of additional signals (e.g., nasopharyngeal pressure) unavailable in the non-invasive setting of the current study. Nevertheless, a decrease in Xrs during expiration (pattern B) is most likely caused by glottic braking that help maintain the end-expiratory lung volume in the early phase of postnatal lung and chest wall development (22). The small but highly significant increase in the T_E_/T_Tot_ ratio in this group (Table 1) supports the above argument. Intuitively, a similar change in Xrs but in inspiration (Pattern C) can be attributed to the negative pressure swings in the glossopharyngeal area, which lead *via* deformation of soft tissues to inspiratory V’ limitation and are augmented by the large nasal component of Rrs. This suggests that the nasal impedance is not only a significant additive component in Zrs (8, 11, 23) but it may modulate the transmural pressures in the compliant structures of distal extrathoracic airways more than in the case of oral breathing. Whereas Patterns B and C describe temporary changes in Xrs, pronounced in mid-expiration or mid-inspiration, respectively (Figure 3), Pattern D is characterized by marked positive increases in Xrs with both inspiratory and expiratory V’. This is likely to be associated with the increased impedance of the nasal pathway, in terms of both resistance and inertance, as reflected by the higher values of R and L in this group (Table 2), also manifested in the significant elevations in zero-V’ Xrs (X_eE_ and X_eI_, Table 3). Note that while the relatively low numbers of Pattern C and Pattern D subjects warrant considerations in their statistical assessments, the frequency of these patterns can be regarded as an inherent feature of the studied healthy term infants. Importantly, Xrs patterns suggesting intrapulmonary expiratory flow limitation observed in the South African cohort of 6-week-old infants (7) were not detected in the present study.

The respiratory pattern can undergo gradual or abrupt changes in a relatively short time (Figure 2). After examination of individual recordings, it can be concluded that a V’-dependent Xrs pattern is not a permanent characteristic of a newborn, but a temporary feature. Sudden changes in the V’-non-linearities might explain the huge day-to-day variability of spectral oscillometry (5). Therefore, measurements of both intra-breath and spectral oscillometry in the same session are recommended to detect and explain the short term changes in respiratory mechanics.

### Within-Session Variability of Oscillometry Measures

The 90-s recordings allowed us to have a closer look into the short-term changes in intra-breath Zrs, which sometimes even disclosed transitions from one pattern of V’ dependence into another. The within-session variability of intra-breath Zrs measures was slightly larger than that of the breathing pattern descriptors, which were obtained from the same recordings (Table 4). Although this may suggest that fluctuations in the spirogram cause changes in the intra-breath parameters, correlation analysis did not confirm such a relationship; similarly, no direct correlations were found between the tidal breathing pattern and the within-session variability of spectral oscillometry (data not reported).

The fact that the most stable spectral measures were R and |Z_8_| is somewhat surprising, as we expected a large variability contributed by the nasal pathway. Explanations based solely on our non-invasive measurement data would be speculative; however, there is indication that the nasal and the distal pulmonary resistances can change in opposite direction to maintain a relatively constant total resistance (23). The highest variability was observed in Ax, which is widely considered as a robust measure of elastic properties of the respiratory system (24). However, as the area of the negative Xrs domain is terminated by f_res_, changes in the dominant nasal inertance would strongly influence the Ax values. Indeed, the intra-breath analysis revealing the patterns of V’ dependence indicated that Ax was biased by changes in Xrs (Table 2), whereas the model fitting of Zrs spectra accounted for the changes in L and resulted in remarkably constant estimates of C for all patterns in spontaneously breathing infants. The model-based approach supported by intra-breath analysis thus makes the values of C less influenced by the strong upper airway compartment and more specific to the elastic properties of the lungs.

### Implications in Oscillometry Procedures in Infants

Technical standards and protocols of spectral oscillometry have been developed for cooperating children and adults (25, 26); these include reproducibility criteria based on repeated measurements that are separated by intervals when the subject is detached from the device. This protocol is clearly impractical to adopt in infant studies, primarily because of the removal and replacement of the face mask may alter the breathing pattern and the sleep stage *via* excitation of the facial nerves (27). Additionally, a minimum of 3 measurement epochs whose lowest-frequency Rrs values have a CoV of ≤ 10% (adults) or ≤ 15% (children) has been suggested as the reproducibility criterion (25). The wide ranges of within-session CoV values of tidal breathing and Zrs parameters observed in the present study (Table 3) may reflect a higher degree of natural variability in respiratory mechanics in neonates (28) compared with older subjects. Therefore, more permissive reproducibility criteria combined with the equally important Xrs measures and based on longer recordings should be established for infants. On the other hand, inclusion of the nasal passages in the infant oscillometry requires careful inspection of the patency of this pathway; congestion may lead to extreme values in R and L and associated with the Xrs pattern D, as in the current measurements.

### Instrumentation Requirements

The spectral measures of Zrs in the present study, as expressed by the R, C and L parameter values (Table 2), are very close to that obtained with the same technique previously (2, 4, 5) and correspond to an impedance magnitude of 40–60 hPa.s.L^–1^. Far above these values representing *averages* for whole breathing cycles, huge *peak values* in Zrs were identified by the intra-breath tracking to occur at instances of V’_maxE_ and V’_maxI_. As illustrated in Figures 2, 3, and quantified by the R_ppI_/Z_0_ and R_ppE_/Z_0_ data in Table 2, Zrs often exceeded 200 hPa.s.L^–1^ in the healthy term newborns of this study. This highlights the need for accurate measurements at peak values of Zrs and not only in intra-breath analysis, as the average values obtained in the conventional spectral oscillometry would also be distorted if an upper range of Zrs is misestimated. The wave-tube principle (29) employed in the present work is particularly advantageous in the measurements of high Zrs, and it was considered as the gold standard technique in the comparison of commercially available oscillometry devices (30). Device dependence might have been the primary reason for the large differences in the Rrs and Xrs values reported recently (13, 31), compared with that from the present and previous measurements with the wave-tube technique (2, 4, 5, 7).

### Implications in Tidal Breathing Analysis

Comparative analysis of tidal breathing and oscillometry indices has revealed generally modest interrelationships (Figure 5) but pinpointed a strong connection between T_PTEF_/T_E_ and an asymmetry measure of V” (V”_eE_/V”_eI_). T_PTEF_/T_E_ can be obtained in relatively simple measurement settings and it has often been considered as a useful index to detect airway obstruction (32–34), although the assessment of T_PTEF_/T_E_ as a surrogate of mechanical tests is controversial in the literature.

In the current study, T_PTEF_/T_E_ did not correlate with the intra-breath Rrs or Xrs variables, and was not different between groups of V’ dependence of Xrs. However, the mean values of V”_eE_/V”_eI_ of the subjects and the corresponding T_PTEF_/T_E_ data covered wide ranges (Figure 6) with a strong linear relationship. This suggests that in healthy term newborns, such as those in the present study, marked differences in the activity of the respiratory control mechanisms rather than airway obstruction exist and determine the values of T_PTEF_/T_E_ (35, 36).

### Limitations

(i) The spectral and intra-breath oscillometry data were derived from recordings collected separately. In order to minimize systematic errors, the two modalities were alternated and, whenever possible, without the removal/repositioning of the face mask. Although there was good agreement in the mean 16-Hz Zrs data collected from the two modalities, the unchanged status of the respiratory mechanical system could not be guaranteed.

(ii) Although sleep state can be an important factor when interpreting lung function measurements in sleeping infants, addressing the relationship between the sleep state and respiratory mechanics was beyond the scope of the current study. Sleep states such as the active (rapid eye movement – REM) sleep and the quiet (non-REM) sleep typically last for 50–70 min in healthy newborns (37), and while we cannot exclude the possibility that a transition between sleep states took place during the measurements, it was more likely that the same state persisted during our recording sessions of typically 14-min duration. Since the estimated ratio of active and quiet sleep is approximately 2:1 in healthy term newborns (37), we can assume that a non-negligible portion of recordings was collected during active sleep. Regularity of the respiratory pattern is also known to be different during active and quiet sleep (38, 39); from the present data it can only be inferred that the variability of T_tot_ and V_T_ was independent of the Xrs pattern of V’ dependence (Table 1).

(iii) The measurement device imposes some impedance against the breathing, which may alter the pattern of tidal breathing without this load. In the present study, the total load including the bacterial filter, the wave-tube, the pneumotachograph and the breathing tube amounted to 6.5 hPa.s.L^–1^, i.e., roughly 10–15% of Rrs. Even if this additional load does not interact with the breathing pattern significantly, it increases the flow-dependent changes in the glossopharyngeal area and may augment the upper airway non-linearities.

## Conclusion

The impedance tracking employed in the present study revealed marked intra-breath changes in Rrs and Xrs in healthy term neonates during natural sleep in the first few days of life. These changes were dominated by the increases in Rrs with V’ in both inspiration and expiration, whereas Xrs exhibited different patterns of change, such as inspiratory and expiratory flow limitation. It is suggested that these intra-breath non-linearities are of upper airway origin, with fundamental contributions from the nasal pathways. Intra-breath changes exert a biasing effect on the conventional measures of the multi-frequency oscillometry that are intended to characterize pulmonary mechanics. It is recommended that the measurements of Zrs in infants cover longer study intervals than that required from cooperative subjects to account for the variable mechanical status of the developing respiratory system. Use of intra-breath oscillometry is proposed to gain more insight into the mechanisms determining Zrs and to properly interpret the results of conventional spectral oscillometry in infants.

## Data Availability Statement

The original contributions presented in the study are included in the article/supplementary material, further inquiries can be directed to the corresponding author.

## Ethics Statement

The studies involving human participants were reviewed and approved by Institutional Clinical Ethics Committee of the University of Szeged (91/2011, renewed in 2017). Written informed consent to participate in this study was provided by the participants’ legal guardian/next of kin.

## Author Contributions

BR, ZGy, and ZH: study design and evaluation of measurements. BR and ZGy: impedance measurements. GM, ZGy, and ZH: design of the infant oscillometry system. BR, GM, ZGy, DC, and ZH: development of the infant intra-breath analysis. BR, DC, and ZH: interpretation of results. BR and ZH: drafting of the manuscript. All authors contributed to the article and approved the submitted version.

## Conflict of Interest

The authors declare that the research was conducted in the absence of any commercial or financial relationships that could be construed as a potential conflict of interest.

## Publisher’s Note

All claims expressed in this article are solely those of the authors and do not necessarily represent those of their affiliated organizations, or those of the publisher, the editors and the reviewers. Any product that may be evaluated in this article, or claim that may be made by its manufacturer, is not guaranteed or endorsed by the publisher.

## Footnotes

1
https://www.rstudio.com
